# Establishing a Reference Interval for Fibroblast Growth Factor (FGF)-23 in Cats

**DOI:** 10.3390/ani14111670

**Published:** 2024-06-03

**Authors:** Sandra Lapsina, Jennifer von Luckner, Nicole Nagler, Simon Franz Müller, Elisabeth Müller, Ingo Schäfer

**Affiliations:** 1 LABOKLIN GmbH and Co. KG, Steubenstraße 4, 97688 Bad Kissingen, Germany; lapsina@laboklin.com (S.L.);; 2 AniCura Ahlen, Bunsenstraße 20, 59229 Ahlen, Germany

**Keywords:** feline, chronic kidney disease, diagnostics, clinical pathology

## Abstract

**Simple Summary:**

Fibroblast growth factor (FGF)-23 is a phosphaturic hormone. The information regarding reference intervals (RIs) of FGF-23 in cats is limited. We aimed to establish RIs in a large cohort of clinically healthy cats and to investigate correlations with sex and age. A total of 118 cats with unremarkable complete blood count and serum chemistry profiles were included. RIs were calculated using the reference interval advisor software 2.1 (Microsoft Excel). The RI for FGF-23 concentrations spanned 85.8 to 387.0 pg/mL (90% confidence interval: lower limit 40.5 to 103.9 pg/mL, upper limit: 354.6 to 425.0 pg/mL). No significant relationships (r^2^ = 0.044) were detected with age (*p* = 0.081) or sex (*p* = 0.191). Other studies of the same diagnostic assay calculated RIs of 56 to 700 pg/mL in 79 cats and <336 pg/mL in 108 cats, and concordance with the present study did not detect any correlation with sex or age.

**Abstract:**

Fibroblast growth factor (FGF)-23 is a phosphaturic hormone. An association between increasing FGF-23 levels and progression of chronic kidney disease (CKD) was documented in cats, dogs, and humans. The information regarding reference intervals (RIs) of FGF-23 in cats is limited. We aimed to establish RIs in a large cohort of clinically healthy cats and to investigate correlations with sex and age. A total of 118 cats with unremarkable complete blood count and serum chemistry profile were included. Clinically sick cats, cats with concurrent diseases, suspicion of CKD, or receiving renal diets were excluded. FGF-23 concentrations were measured with the FGF-23 ELISA Kit. RIs were calculated using the reference interval advisor software 2.1 (Microsoft Excel). FGF-23 concentrations were correlated with sex and age. The RI for FGF-23 concentrations spanned 85.8 to 387.0 pg/mL (90% confidence interval: lower limit 40.5 to 103.9 pg/mL, upper limit: 354.6 to 425.0 pg/mL). No significant relationships (r^2^ = 0.044) were detected with age (*p* = 0.081) or sex (*p* = 0.191). Other studies of the same diagnostic assay calculated RIs of 56 to 700 pg/mL in 79 cats and <336 pg/mL in 108 cats, and in concordance with the present study, did not detect any correlation with sex or age.

## 1. Introduction

Chronic kidney disease (CKD) is one of the most common diseases in cats with an overall prevalence of 2 to 4% [1,2]. In cats over 10 years of age, the prevalence can be as high as 30 to 40% [3,4]. The prognosis correlates with the CKD stage as defined by the International Renal Interest Society (IRIS). Staging is based on such parameters as serum creatinine concentration, blood pressure measurements, and urine protein/creatinine ratio [5]. The modified IRIS guidelines also include concentration of serum symmetric dimethylarginine (SDMA) as a marker for glomerular filtration rate (GFR) [6]. Moreover, Fibroblast Growth Factor (FGF)-23 has also recently been added to the IRIS guidelines as a marker for deranged metabolism.

FGF-23 is a phosphaturic hormone produced by osteocytes and osteoblasts. It is responsible for the homeostasis of phosphorus and calcitriol [7,8]. FGF-23 regulates serum phosphate levels by increasing the excretion of renal phosphate and decreasing the synthesis and degradation of calcitriol. An increase in FGF-23 concentration promotes urinary excretion of phosphorus and decreases its absorption in the intestines [9,10,11]. In addition, FGF-23 also acts on the parathyroid gland and reduces both the synthesis and secretion of the parathyroid hormone [12]. In humans suffering from CKD, serum FGF-23 concentration rises exponentially as renal function declines, far preceding any significant increase in serum phosphate or parathyroid hormone concentrations [12,13,14]. Additionally, FGF-23 concentration negatively correlates with the estimated GFR in humans [14]. Hyperphosphatemia and increased calcitriol concentration in blood both stimulate FGF-23 production.

So far, several studies have indicated FGF-23 as a promising early parameter for phosphate derangement in feline CKD [15,16,17,18]. FGF-23 might also have diagnostic relevance for the early detection of both CKD and phosphate derangement in cats with CKD. Disruption of phosphate homeostasis may develop in the early stages of CKD, preceding azotemia or hyperphosphatemia. FGF-23 concentration is significantly higher in cats with azotemic CKD when compared to healthy animals and increases significantly with the progression of CKD [15,16]. However, the true diagnostic potential of FGF-23 in cats remains largely unknown.

Regarding the use of available FGF-23 assays in cats, several studies have been performed. The following diagnostic quantitative enzyme-linked immunosorbent assay (ELISA) kits have been tested: the FGF-23 ELISA kit (Kainos Laboratories, Tokyo, Japan) by four studies [15,17,19,20], the MyBioSource ELISA (MyBioSource, San Diego, CA, USA) by two studies [16,21], and the MedFrontier FGF23 ELISA (Minaris Medical Co., Tokyo, Japan) by another study [22]. In a study from 2023 performed by the same authors, where the FGF-23 ELISA kit, the MyBioSource ELISA, and the LIAISON FGF 23 kit for intact FGF-23 were all compared on the Liaison platform developed by DiaSorin (Saluggia, Italy), the FGF-23 ELISA kit (Kainos Laboratories, Tokyo, Japan) demonstrated the best correlation with various creatinine concentrations according to the IRIS [23]. The intra- and interassay coefficients of variation (CVs) of this assay were all below 15%, thus indicating a reliable performance [23].

Nevertheless, all current knowledge regarding reference intervals (RIs) for the FGF-23 ELISA kit is derived from only two studies, providing the following RIs of 56 to 700 pg/mL in 79 cats [15] and 0 to 366 pg/mL in 108 cats [20]. Therefore, the aim of this study was (1) to establish RIs for FGF-23 in a large cohort of clinically healthy cats and (2) to investigate correlations of FGF-23 concentration with sex and age in clinically healthy, non-azotemic cats.

## 2. Materials and Methods

### 2.1. Study Population

The submitting veterinarians were asked to provide information on the general health status of the individual cats at the time of blood collection, any concurrent acute as well as chronic diseases, including suspicion for CKD, and administration of renal diet. This data was collected by questionnaires and telephone calls. Inclusion criteria for the cats were: classified as clinically healthy by the treating veterinarian, no known acute or chronic diseases, no present clinical signs typical of CKD, and not receiving any specific renal diet.

### 2.2. Sample Collection

Serum samples used in this study were leftover volumes from the send-in routine diagnostic samples of the clinical laboratory LABOKLIN (Bad Kissingen, Germany) from December 2022. Only cats with unremarkable Complete Blood Count (CBC) results performed by the Sysmex XN-V analyzer (Sysmex Deutschland, Norderstedt, Germany) on EDTA blood, as well as unremarkable biochemistry profile findings performed by Cobas 8000 (Roche, Mannheim, Germany: alanine transaminase, albumin, alkaline phosphatase, alpha-amylase, bilirubin, calcium, cholesterol, creatine kinase, DGGR-lipase, fructosamine, globulin, glucose, glutamate dehydrogenase, magnesium, creatinine, SDMA, phosphorus, potassium, sodium, total protein, triglycerides, and urea) on the serum samples according to the reference intervals of the laboratory were included in this study. Only cats with creatinine concentration of <140 µmol/L (1.6 mg/dL) and SDMA concentration of <18 µg/dL were included, which corresponds to the IRIS stage 1. Hematological and the above-mentioned biochemical parameters were evaluated immediately after an overnight shipment to the laboratory. Meanwhile, for the FGF-23 measurements, the serum samples were stored frozen for a maximum of 5 working days at −20 °C.

### 2.3. Analytic Methods

FGF-23 concentration was measured in serum with the FGF-23 ELISA Kit (Kainos Laboratories, Tokyo, Japan) according to the manufacturer’s guidelines. According to the manufacturer’s specifications, the minimum detection limit for the Kainos ELISA assay was 3 pg/mL, while the quantification range spanned 3–800 pg/mL. In the LABOKLIN laboratory, coefficients of variation of <15% are considered to indicate a good performance of the chosen diagnostic assay in cats [23]. In detail, the intra-assay precision in the LABOKLIN laboratory showed coefficients of variation of 12.54%, 4.17%, and 4.19%, while inter-assay precision values were 9.2%, 9.96%, and 8.17% for samples with low, moderate, and high FGF-23 levels, respectively [23].

### 2.4. Statistical Analysis

Age and sex were analyzed descriptively. Statistical analysis was completed using SPSS for Windows (version 29.0; SPSS, Armonk, NY, USA). The Anderson–Darling test was used to check for normality. Reference intervals for FGF-23 were calculated nonparametrically according to the ASVCP reference interval guidelines [24] using the reference interval advisor software 2.1 on Microsoft Excel for Microsoft Windows 365 [25] and comprised the central 95% of the fitted distribution with 90% confidence intervals calculated around the lower (2.5%) and upper (97.5%) limits using the bootstrap method. Prior transformation of the reference data was done and Horn’s algorithm using Tukey’s interquartile fences were used for identification of potential outliers. A multiple linear regression model of FGF-23 concentrations was fitted with age and sex as cofactors. The Mann–Whitney U-test was used for comparison of FGF-23 levels regarding sex and age. Statistical significance was set at *p* < 0.05.

## 3. Results

### 3.1. Study Population

The age and sex were known in all 118 cats included in this study. Regarding age, a median age of 9 years was calculated (mean 8.81 years, standard deviation 4.36 years, minimum 1 year, maximum 20 years). Regarding sex, 55/118 cats were male (46.6%, intact 10/55 cats [18.2%], castrated 45/55 cats [81.8%]) and 63 females (53.4%, intact 14/63 cats [22.2%], spayed 49/63 cats [77.8%]).

### 3.2. FGF-23 Concentrations

The serum FGF-concentrations were not normally distributed (Anderson–Darling testing: *p* = 0.004) in this population of 118 cats and ranged from 40.5 to 425.0 pg/mL (mean: 210.2 pg/mL, median 204.8 pg/mL, standard deviation 77.8 pg/mL).

In female cats, FGF-23 showed a median of 210.2 pg/mL (mean 219.9 pg/mL, standard deviation 79.9 pg/mL, minimum 40.5 pg/mL, maximum 425.0 pg/mL). A median of 179.6 pg/mL was demonstrated in males (mean 199.1 pg/mL, standard deviation 74.4 pg/mL, minimum 86.2 pg/mL, maximum 398.7 pg/mL). No statistically significant differences in FGF-23 levels were demonstrated comparing male to female cats (*p* = 0.102).

The study group was divided into cats < 9 years (*n* = 52) and cats ≥ 9 years (*n* = 66) according to the median of 9 years in the cats included in the study. In cats < 9 years, FGF-23 levels showed a median of 177.8 pg/mL (mean 194.3 pg/mL, standard deviation 80.4 pg/mL, minimum 40.5 pg/mL, maximum 386.7 pg/mL) compared to a median of 212.6 pg/mL (mean 222.7 pg/mL, standard deviation 73.8 pg/mL, minimum 99.6 pg/mL, maximum 425.0 pg/mL) in cats ≥ 9 years. There was a statistically significant impact of age on FGF-23 levels (*p* = 0.025).

### 3.3. FGF-23 Reference Interval

**Figure 1:**
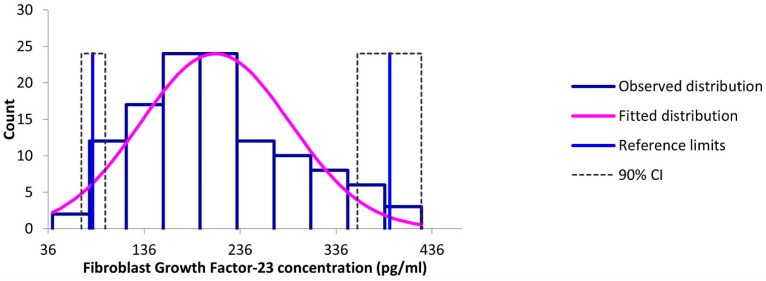
Distribution of Fibroblast Growth Factor-23 concentrations in 118 clinically healthy cats.

No outliers were identified by the Tukey method. The RI for FGF-23 concentrations spanned between 85.8 and 387.0 pg/mL with a 90% confidence interval of 40.5 to 103.9 pg/mL for the lower limits and 354.6 to 425.0 pg/mL for the upper limits (Figure 1). A generalized linear model did not detect any significant correlation (r^2^ = 0.044) between serum FGF-23 concentrations and age (*p* = 0.081) or sex (*p* = 0.191).

## 4. Discussion

The FGF-23 ELISA Kit (Kainos Laboratories, Tokyo, Japan) demonstrated the best diagnostic performance in a study by the same authors in 2023 when three different FGF-23 assays were compared in cats [23]. Although originally developed to detect human FGF-23, this assay was soon successfully applied in cats as well [15,17,23]. The knowledge regarding RIs for this FGF-23 ELISA kit is currently based on two studies demonstrating RIs of 56 to 700 pg/mL in 79 cats [15] and 0 to 366 pg/mL in 108 cats [20]. The calculated RI in our study (85.8 to 387.0 pg/mL) is in concordance with the second study, especially regarding the upper limit. Regarding the FGF-23 measurements, it must also be mentioned that assay- and species-specific RI seem to be important. Thus, the study comparing three different diagnostic FGF-23 assays revealed a high variation of FGF-23 concentrations in cats depending on the assay used [23]. Meanwhile, in a study performed by the same authors in clinically healthy dogs (*n* = 136) with the same FGF-23 assay, a RI of 95.8 (90% confidence interval: 44.6–139.2 pg/mL) to 695.1 pg/mL (90% confidence interval: 598.7–799.1 pg/mL) was established and was thus much wider than the RI currently established in cats [26]. Moreover, the upper RI in 115 clinically healthy pediatric human subjects was 61.2 pg/mL (90% confidence interval: 58.6–63.7 pg/mL) [27] using the LIAISON FGF 23 kit for intact FGF-23 on the Liaison platform developed by DiaSorin (Saluggia, Italy), further emphasizing the importance of species-specific and assay-related RIs.

The storage of a maximum of five days while frozen most likely did not influence the FGF-23 concentrations in the samples of our study, as FGF-23 has been documented to show no significant changes in samples stored for various periods at different temperatures in earlier studies [28,29]. The general guidelines of the laboratory recommend taking blood samples after overnight fasting to exclude the influence of feeding, especially on biochemistry parameters.

FGF-23 is a useful biomarker for the identification and better understanding of CKD–mineral and bone disorder [30]. Further studies in cats are needed to evaluate the outcome of an early dietary invention in cats with increased FGF-23 levels. The value of FGF-23 in the identification and prediction of CKD progression has to be further evaluated [30].

Regarding age, a statistically significant difference in FGF-23 concentrations was noted with clinically healthy dogs aged < 9 years to dogs 9 years of age or older with a trend for increasing FGF-23 concentrations with age [26]; it was also seen in cats in our study as well as in a previous study [17]. However, no significant correlation of FGF-23 concentrations with age or sex was demonstrated. In a study with clinically healthy geriatric cats, FGF-23 concentrations of up to 700 pg/mL were detected [15], which was not seen in our study. However, in the 11 cats aged 15 years and older in this study, a median FGF-concentration of 253.1 pg/mL was noted (mean 236.3 pg/mL, standard deviation 56.5 pg/mL, minimum 104.0 pg/mL, maximum 313.8 pg/mL), which was higher than the results of the whole population altogether (mean: 210.2 pg/mL, median 204.8 pg/mL, standard deviation 77.8 pg/mL). Further studies with larger cohorts and a higher number of clinically healthy geriatric cats are recommended.

### Limitations

For cats included in calculations for RIs, health status was based both on the results of questionnaires to the veterinarians and on unremarkable hematological and biochemical findings. Potentially important background information was unavailable to the authors, including living conditions and reasons for blood sampling and laboratory testing. Therefore, no data regarding history, clinical signs, blood pressure measurement, urinalysis, diagnostic imaging, and prior or current medications were included in the study. The application of phosphate binders could also not be ruled out with certainty. Due to the missing urinalysis, the measurement of the specific urinary gravity in the cats included in the study and substaging using the urinary protein-to-creatinine-ratio to rule out proteinuria as recommended by the IRIS guidelines [6] was not possible. Therefore, any tubular dysfunction may have been missed, potentially influencing the FGF-23 production by renal tubules in the cats included in the study.

## 5. Conclusions

An RI for FGF-23 concentrations of 85.8 to 387.0 pg/mL with a 90% confidence interval of 40.5 to 103.9 pg/mL for the lower limits and 354.6 to 425.0 pg/mL for the upper limits was established in a population of 118 clinically healthy cats. This is in accordance with other studies that have calculated the RIs to be 56 to 700 pg/mL and 0 to 336 pg/mL. The latter study detected no statistical impact of sex and age on FGF-23 concentrations, which agreed with our study. Larger study cohorts of clinically healthy cats are further recommended.

## Data Availability

The original contributions presented in the study are included in the article, further inquiries can be directed to the corresponding author.
